# Supplementary material for: pH and Redox-Dual Sensitive Chitosan Nanoparticles Having Methyl Ester and Disulfide Linkages for Drug Targeting against Cholangiocarcinoma Cells
Source: Materials (Basel). 2022 May 26;15(11):3795. doi: 10.3390/ma15113795 (PMC9181436; doi:10.3390/ma15113795)
Supplement: Supplementary file 1 [file materials-15-03795-s001.zip › materials-1725089-supplementary.pdf]

Supporting Materials

Experimental

Analysis of Particle size distribution

Zetasizer Nano-ZS® (Malvern, Worcestershire, UK) was employed to measure particle size distribution. The nanoparticle concentration in the distilled water was adjusted to 0.1 % (w/w) and measured at 20 °C.

Results

As shown in Figure S1, TEM photo indicated that ChitoHISss nanoparticles have spherical shapes and their particle sizes were less than 200 nm. Average particle size of ChitoHISss nanoparticles was 134.5±18.4 nm. Their average particle sizes were almost similar to the results of the particle size analysis results as shown in Figure 2 and Table 1.

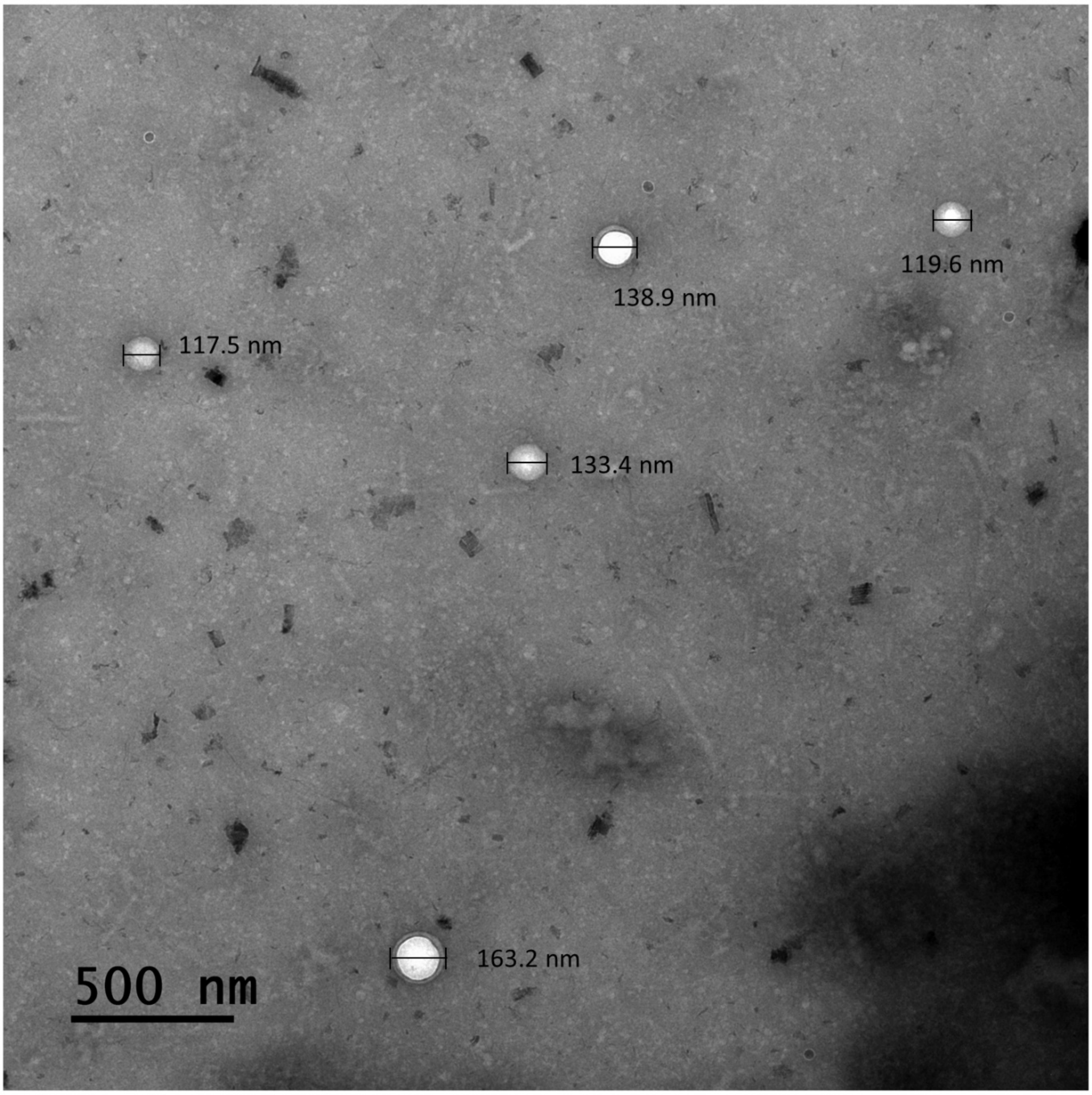

Figure S1. Morphological observation of ChitoHISss nanoparticles and their particle size. Particle size indicated in the photo was estimated by photo of TEM equipment.
